# Thrombophilic Risk of Factor V Leiden, Prothrombin G20210A, MTHFR, and Calreticulin Mutations in Essential Thrombocythemia Egyptian Patients

**DOI:** 10.1155/2020/7695129

**Published:** 2020-03-30

**Authors:** Mohamed S. El-Ghonemy, Solafa El Sharawy, Maryan Waheeb Fahmi, Shaimaa El-Ashwah, May Denewer, MA El-Baiomy

**Affiliations:** ^1^Hematology Unit, Clinical Pathology Department, Mansoura University Faculty of Medicine, Mansoura, Egypt; ^2^Medical Oncology Unit, Oncology Center, Mansoura University, Faculty of Medicine, Mansoura, Egypt; ^3^Clinical Hematology Unit, Oncology Center, Mansoura University, Faculty of Medicine, Mansoura, Egypt

## Abstract

**Objectives:**

Essential thrombocythemia (ET) is one of the myeloproliferative neoplasms characterized by a sustained elevation of platelet numbers with a tendency for thrombosis and hemorrhage. The aim of this work is to establish the relation between calreticulin, factor V Leiden, prothrombin G20210A, and MTHFR mutations in ET patients and the thrombotic risk of these patients.

**Methods:**

This study was carried out on 120 ET patients and 40 apparently healthy individuals as a control group.

**Results:**

There were increases in WBCs, PLT counts, PT, fibrinogen concentration factor V Leiden, and MTHFR mutation in ET patients as compared to the control group (*P* < 0.05). Also, there were increases in WBCs, PLT counts, and hematocrit value in thrombosed ET patients as compared to the nonthrombosed ones (*P* < 0.05). On the contrary, there was no significantly statistical difference in ET patients with JAK2 V617F positive mutation versus the JAK2 negative group (*P* > 0.05) and in patients with cardiovascular risk factors versus patients with noncardiovascular risk factors (*P* > 0.05). ET patients with factor V Leiden, prothrombin gene, and CALR mutations were more prone to thrombosis (odds ratio 5.6, 5.7 and 4.7, respectively). On the contrary, JAk2V 617F and MTHFR mutations have no effect on the thrombotic state of those patients.

**Conclusion:**

There is a significant increase risk of thrombosis in ET patients with CALR mutation, thrombophilic mutations, as well as factor V Leiden and prothrombin gene mutation with a risk of developing leukemic transformation.

## 1. Introduction

Essential thrombocythemia (ET) is one of the “myeloproliferative neoplasms” (MPNs) characterized by clonal overproduction of one or more blood cell lines. ET is characterized by a sustained elevation of platelet number with a tendency for thrombosis and hemorrhage [1]. ET may be asymptomatic or it may be presented by vascular occlusive events and hemorrhages [1, 2]. The most common molecular marker of these diseases is JAK2 V617F mutation present in 50–70% and calreticulin (CALR), a multifunctional calcium-binding protein, mutation present in 15–24% [1].

Thrombophilia is common in ET patients which may be genetic or acquired. The pathogenesis of thrombosis in ET is complex and not yet fully understood [2]. Several defects have been described, including an autonomous production of platelets unregulated by the physiologic feedback mechanism to keep the count within the reference range [3]. Also, in ET, there were increased sensitivities to cytokines (e.g., interleukin-3 (IL-3)) and decreased inhibition to platelet-inhibiting factors (e.g., transforming growth factor (TGF) beta), leading to hyperaggregation [4]. However, Pearson [5] reported that platelet count per se does not correlate with thrombotic incidence.

Thrombophilic genes in the form of factor V Leiden is a mutation in the factor V gene accounting for 40–50% of thrombosis [6]. Prothrombin G20210A is the second most prevalent genetic abnormality causing thrombophilia [6, 7]. Methylene tetrahydrofolate reductase (MTHFR) plays a major role in folate metabolism [8, 9]. Disturbed function of the enzyme results in hyperhomocysteinemia and may cause thrombophilia [7, 10].

To our knowledge, the relation between ET and factor V Leiden, prothrombin G20210A, and MTHFR gene mutations is not yet fully understood. So, the aim of this work is to establish the relation between calreticulin, factor V Leiden, prothrombin G20210A, and MTHFR mutations in ET patients and the thrombotic risk of these patients, as independent factors or as covariates with other biological features.

## 2. Patients and Methods

A case control study was conducted on 160 individuals: 120 patients (51 males and 69 females) ranging between 17 and 86 years old (mean age 47.7) and 40 apparently healthy individuals of matched age and sex with the mean age of 45.1 years, who served as the control group. The patients were diagnosed based on the WHO 2016 criteria for diagnosis of ET [1] and were admitted to Mansoura oncology center.

All patients gave informed consent for both treatment and genetic analysis. The samples were taken at time of diagnosis, and the patients were followed up for 24 months. Forty-two patients developed thrombosis: 30 patients at the time of diagnosis and 12 during the follow-up period. Forty patients had venous thrombosis, and 2 had arterial thrombosis. According to the presence of JAK2 mutation, the patients divided into 78 JAK2 positive patients and 42 JAK2 negative patients; according to the presence of CALR mutation, the patients divided into 14 CALR positive patients and 106 CALR negative patients, and according to CVS risk factors (smoking, HTN, DM, and hyperlipidemia), the patients were divided into 66 high-risk patients and 54 without risk factors. The patients and the control group were subjected to history taking, clinical examination, complete blood count (CBC), bleeding time, prothrombin time (PT), activated partial thromboplastin time (APTT), fibrinogen concentration using the coagulation technique, Va1617Phe JAK-2 mutation, calreticulin mutation, and the polymorphism of factor V Leiden, prothrombin G20210A, and MTHFR mutation gens.

The patients were treated with low-dose aspirin (ASA, 40 to 100 mg/day PO). Hydroxyurea (initial dose 15 mg/kg per day PO, taken in divided doses and maintenance doses adjusted up or down depending upon the balance between the desired effect on the platelet count, which should be kept in the range of 100,000 to 400,000/microL, and undesired effects such as neutropenia and anemia). The aims of the treatment in those patients were to minimize the abnormal clinical, physical, and laboratory features while also minimizing the long-term complications of the disease (e.g., thrombotic events, bleeding, myelofibrosis, acute leukemia, and other malignancies).

### 2.1. PCR Detection of the JAK-2 V617F Mutation Using ASO-Specific PCR Genetic Marker

The detection of JAK2 V617F mutation was mutation by using the following primers (JAK2 reverse: 5′ CTGAATAGTCCTACAGTGTTTTCAG‐TTTCA 3′, JAK2 forward (specific): 5′ AGCATTTGGTTTTAAATTATGGAGTATATT 3′, and JAK2 forward (internal control): 5′ ATCTATAGTCATGCTGAAAGTA‐GGAGAAAG 3′). Cycling conditions were 35 cycles with annealing temperature 58.5°C followed by agarose gel 2% electrophoresis for band detection [11].

### 2.2. PCR Detection of the Calreticulin Mutation

The detection of calreticulin mutation was performed by using the following primers (CALR forward: 5′ TAACAAAGG‐TGAGGCCTGGT 3′ and CALR reverse: 5′ GCCTCTCT‐ACAGCTCGTCCTT 3′). Cycling conditions were 35 cycles with annealing temperature 58.5°C followed by cycle sequencing of PCR products with the respective forward and reverse primer using an automated ABI 310 DNA sequencer [12].

### 2.3. Factor V Leiden, Prothrombin G20210A, and MTHFR Mutation Detection

DNA extraction was performed for all studied samples followed by PCR amplification using biotinylated primers supplied by the kit (FV-PTH-MTHFR Strip Assay, Vienna Lab, Austria, http://www.viennalab.com). The PCR products were hybridized to a test strip containing allele-specific oligonucleotide probes immobilized as an array of parallel lines [13].

## 3. Results

The present study were conducted on 160 individuals; 120 patients (51 males and 69 females), and 40 apparently healthy individuals of ages ranging between 25 and 60 years old, who served as the control group.

Regarding the haemogram and coagulation profile tests, there were significant statistical increase of WBCs, PLT counts, prothrombin time, and fibrinogen concentration in the patients group as compared with the control groups (*P* < 0.05) (Table 1). Also, there was significant statistical increase of WBCs, PLT counts, and hematocrit value in thrombosed patients as compared to nonthrombotic patients (*P* < 0.05) (Table 2). On the contrary, there was no significantly statistical difference, regarding unthrombosed compared to those developed thrombosis, in ET patients with JAK2 V617F positive mutation versus the JAK2 negative group (*P* > 0.05) or patients with cardiovascular risk factors versus patients with noncardiovascular risk factors (*P* > 0.05) (Table 3).

Regarding factor V Leiden, prothrombin G20210A, and MTHFR mutation, there is significant statistical increase in the percentage of factor V Leiden and MTHFR mutation in ET patients compared to the control group (Table 1). Multivariate analysis revealed significant increase the risk of thrombosis in ET patients with factor V Leiden and prothrombin gene mutations (odds ratio 5.6 and 5.7, respectively) (Table 3). On the contrary, no significant statistical difference in the percentage of factor V Leiden, prothrombin gene, and MTHFR mutations in ET patients with JAK2 V617F positive mutation versus the JAK2 V617F negative group (*P* > 0.05) (Table 4) and Percentage of factor V Leiden, prothrombin gene, and MTHFR mutations in patients with cardiovascular risk factors versus the patients with noncardiovascular risk factors group (*P* > 0.05) (Table 5). Six patients developed leukemic transformation during follow-up period: four of them were factor V Leiden and two were prothrombin gene mutant, and the six patients had thrombotic events.

## 4. Discussion

Essential thrombocythemia (ET) is a myeloproliferative neoplasm. It is characterized by increased blood platelets that predispose to multiple vascular events [14]. The disease incidence is estimated to be around 0.01–2.61 per 100,000 with a female predominance and a median age of 71 years old at diagnosis as reported by Titmarsh et al. [15].

With respect to the laboratory data, total leucocyte counts, prothrombin time, and fibrinogen concentration were significantly increased in ET patients compared to the control group (*P* < 0.005). These coincide with those reported by Treliński et al. [16] Comparison between thrombotic ET patients and nonthrombotic group revealed that WBCs counts and platelets counts were significantly increased in thrombotic patients compared to the nonthrombotic group (*P* < 0.05). This was in accordance with those recorded by Carobbio et al. [17] and Lim et al. [18] The leukocytes contribute to the pathogenesis of thrombosis in ET patients through the mechanisms of activation and interaction with platelets and endothelial cells as the activated leukocytes may release proteases and oxygen radicals which alter endothelial cells and platelets. Also, a series of markers of leukocyte activation including expression of membrane CD11B and leukocyte alkaline phosphatase antigen, cellular elastase contents, plasma elastase levels, and myeloperoxidase levels are elevated, and those contributed to establish the prothrombotic state [17, 19]. In addition, in ET patients, there are abnormalities of megakaryocytes being more sensitive to growth factors. These abnormalities not only resulting in elevated platelet count but also the platelets derived from these abnormal megakaryocytes are active and have abnormal function [19].

In this study, JAK2 V617F mutation was detected in 78/120 patients (65.0%). This is in agreement with the study of Palandri et al. [20] which diagnosed the mutation in (61.0%) of ET patients. CALR mutation was detected in 14/120 patients (11.66%) [19]. This is in agreement with the study of Rumi et al. [10] which diagnosed the mutation in (11.4%) of ET patients. Also, cardiovascular risk factors were reported in 66/120 patients (55.0%) with no significant difference regarding the haemogram and coagulation profiles in patients with cardiovascular risk factors compared with those patients of noncardiovascular risk factors.

Considered the mutant genes, the mutant factor V Leiden, prothrombin gene, and MTHFR gene were found in (45.0%, 22.5% and 77.5%, respectively). In this study, there is no significant statistical difference regarding the percentage of those mutant genes in ET patients with JAK2 V617F positive mutation versus the JAK2 V617F negative group, and also there is no significant statistical difference regarding the percentage of those mutant genes in patients with CVS risk factors versus the patients with non-CVS risk factors.

Multivariate analysis regarding the thrombotic state revealed that factor V leiden and prothrombin gene mutations associated with increased risk of thrombosis in ET patients with odds ratio equal to 5.625 and 5.75, respectively. This is in agreement with other studies [21, 22] which observed a significant increase of this mutation in ET patients with thrombotic events. On the contrary, Amitrano et al. [9] and Kornblihtt et al. [23] failed to demonstrate a link between thrombophilic mutations and thrombosis in ET patients. In this study, MTHFR gene mutation was associated with low risk for thrombosis in ET patients with odds ratio equal to 0.595, (*P*=0.1) which is in accordance with the study of Trifa et al. [19]. The explanation of this is due to the food supplementation with folic acid which prevents the development of venous thrombosis [24].

In this study, JAK2 V617F mutation was associated with low risk for thrombosis in ET patients with odds ratio equal to 0.953 (*P*=0.9). On the contrary, Horvat et al. [19] and Fu et al. [25] revealed that JAK2 V617F mutation was associated mainly with arterial thrombosis in ET patients. This difference could be attributed to most of the thrombotic attacks in our study of venous type.

It was observed that, the incidence of thrombosis among ET patients with CALR mutation was 7.5% (*P*=0.04). This is in agreement with other studies which found that the incidence of thrombosis was 14.5% in JAK2 positive and 5% in CALR positive patients [26].

Also, in this study, thrombotic events were reported in 42/120 patients (35.0%), a figure very close to that reported by Lekovic et al. [27]. We also noted that the cardiovascular risk factors are associated with increased risk of thrombosis in ET patients with odds ratio equal to 2.9 (*P* = 0.1). This is in agreement with Lekovic et al. [27] and Montanaro et al. [28].

In conclusion, there is a significant increase risk of thrombosis in ET patients with factor V Leiden, prothrombin gene mutation, and CALR mutation with a risk of developing leukemic transformation. Most of the thrombotic attach in Egyptian ET patients were of venous type. So, further studies on large numbers of patients are needed to validate the value of more antithrombotic prophylaxis with these mutations and to consider these mutations as prognostic molecular markers for ET patients.

## Figures and Tables

**Table 1:** Statistical analysis of haemogram, coagulation profile tests, and thrombophilia gene in essential thrombocythemia patients versus control groups.

	Type	Mean ± SD or *n* (%)	*P*
HG (gm/dl)	Control (40)	11.800 ± 1.4571	0.055
	Patients (120)	10.614 ± 2.4959	

Hematocrit (%)	Control (40)	41.8000 ± 3.41205	0.17
	Patients (120)	38.8250 ± 9.41871	

WBCs (×10^9^/L)	Control (40)	6.200 ± 1.2424	0.001
	Patients (120)	10.883 ± 4.9132	

PLT (×10^9^/L)	Control (40)	262.25 ± 93.346	0.001
	Patients (120)	991.75 ± 428.410	

PT (sec)	Control (40)	11.510 ± 0.9856	0.003
	Patients (120)	12.600 ± 0.3097	

APTT (sec)	Control (40)	32.40 ± 2.722	0.06
	Patients (120)	30.25 ± 4.754	

Fibrinogen concentration (mg/dl)	Control (40)	257.60 ± 6.7	0.001
	Patients (120)	378.13 ± 35.766	

Factor V Leiden (Heterozygous)	Control (40)	4 (10)	0.007
	Patients (120)	54 (45)	

Prothrombin gene mutation (heterozygous)	Control (40)	8 (20)	0.8
	Patients (120)	27 (22.5)	

MTHFR mutation	Control (40)	10 (25)	<0.001
	Patients (120)	93 (77.5)	

**Table 2:** Statistical analysis of patients' haemogram and coagulation profile tests in unthrombosed compared to those developed thrombosis.

	Thrombosis	Mean ± SD or *n* (%)	*P*
HG (g/dl)	No thrombosis (N: 78)	10.142 ± 2.5939	0.1
	Thrombosis (N: 42)	11.491 ± 2.1151	

Hematocrit (%)	No thrombosis (N: 78)	36.5000 ± 9.36910	0.03
	Thrombosis (N: 42)	43.1429 ± 8.15105	

WBCs (×10^9^/L)	No thrombosis (N: 78)	8.296 ± 2.9925	0.01
	Thrombosis (N: 42)	12.687 ± 5.4472	

PLT (×10^9^/L)	No thrombosis (N: 78)	886.38 ± 291.919	0.03
	Thrombosis (N: 42)	1187.43 ± 568.584	

PT (sec)	No thrombosis (N: 78)	12.612 ± 0.3179	0.7
	Thrombosis (N: 42)	12.579 ± 0.3043	

APTT (sec)	No thrombosis (N: 78)	29.96 ± 2.236	0.6
	Thrombosis (N: 42)	30.79 ± 7.597	

Fibrinogen concentration (mg/dl)	No thrombosis (N: 78)	279.19 ± 33.223	0.8
	Thrombosis (N: 42)	276.14 ± 41.333	

**Table 3:** Multivariate analysis of thrombotic risk of ET patients.

	Thrombosis		Odds ratio (confidence interval)	*P*
	Present	Absent		
Factor V Leiden	30 (25	24 (20%)	5.625 (2.3–13.9)	0.014
Prothrombin gene mutation	18 (15%)	9 (7.5%)	5.750 (3.1–15.8)	0.02
MTHFR mutation	30 (25%)	63 (52.5%)	0.595 (1.0–2.5)	0.1
JAK2 V617F mutation	27 (22.5%)	51 (42.5%)	0.95 (0.6–2.8)	0.9
CALR mutation	9 (7.5%)	5 (4.1%)	4.7 (2.9–10.9)	0.04
CVS risk factors (at least one)	36 (30%)	30 (25%)	2.9 (1.1–5.1)	0.1

**Table 4:** Percentage of factor V Leiden, prothrombin gene, and MTHFR mutations in essential thrombocythemia patients with JAK2 V617F positive mutation versus the JAK2 V617F negative group.

		Jak2 V617F mutation				*P*
		Positive mutation		Negative mutation		
		Count	%	Count	%	
Factor V Leiden	Normal	39	59.1	27	40.9	0.3
	Heterozygous	39	72.2	15	27.8	

Prothrombin gene mutation	Normal	60	64.5	33	35.5	0.9
	Heterozygous	18	66.7	9	33.3	

MTHFR mutation	Normal	21	77.8	6	22.2	0.4
	Heterozygous	48	76.2	15	23.8	
	Homozygous	9	30.0	21	70.0	

**Table 5:** Percentage of factor V Leiden, prothrombin gene, and MTHFR mutations in patients with cardiovascular risk factors versus the patients with noncardiovascular risk factors group.

		CVS risk factors				*P*
		With risk factors		Without risk factors		
		Count	%	Count	%	
Factor V Leiden	Normal	39	59.1	27	40.9	0.5
	Heterozygous	27	50.0	27	50.0	

Prothrombin gene mutation	Normal	48	51.6	45	48.4	0.4
	Heterozygous	18	66.7	9	33.3	

MTHFR mutation	Normal	21	77.8	6	22.2	0.6
	Heterozygous	33	52.4	30	47.6	
	Homozygous	12	40.0	18	60.0	

## Data Availability

The data used to support the findings of this study are available from the corresponding author upon request
